# Membrane fusogenic lysine type lipid assemblies possess enhanced NLRP3 inflammasome activation potency

**DOI:** 10.1016/j.bbrep.2019.100623

**Published:** 2019-04-12

**Authors:** Jieyan He, Tianshu Li, Tomasz Próchnicki, Gabor Horvath, Eicke Latz, Shinji Takeoka

**Affiliations:** a Department of Life Science and Medical Bioscience, Graduate School of Advanced Science and Engineering, Waseda University, Tokyo, Japan; b Institute for Advanced Research of Biosystem Dynamics, Waseda Research Institute for Science and Engineering, Waseda University, Tokyo, Japan; c Institute of Innate Immunity, Biomedical Center, University Hospitals, University of Bonn, Bonn, Germany

**Keywords:** Membrane fusion, Endocytosis, Lysine, Cationic liposome, NLRP3 inflammasome, IL-1β

## Abstract

Lysine (K) type cationic lipid with a propyl spacer and ditetradecyl hydrophobic moieties composing liposomes, K3C14, previously studied for gene delivery, were reported to activate the NLRP3 inflammasomes in human macrophages via the conventional phagolysosomal pathway. In this study, K3C16, a propyl spacer bearing lysine type lipids with dihexadecyl moieties (an extension of two hydrocarbon tail length) were compared with K3C14 as liposomes. Such a small change in tail length did not alter the physical properties such as size distribution, zeta potential and polydispersity index (PDI). The NLRP3 activation potency of K3C16 was shown to be 1.5-fold higher. Yet, the toxicity was minimal, whereas K3C14 has shown to cause significant cell death after 24 h incubation. Even in the presence of endocytosis inhibitors, cytochalasin D or dynasore, K3C16 continued to activate the NLRP3 inflammasomes and to induce IL-1β release. To our surprise, K3C16 liposomes were confirmed to fuse with the plasma membrane of human macrophages and CHO-K1 cells. It is demonstrated that the change in hydrophobic tail length by two hydrocarbons drastically changed a cellular entry route and potency in activating the NLRP3 inflammasomes.

## Highlights

• Extension of two hydrocarbon (C16) in hydrophobic tails does not alter physical characters of lysine-type (K) liposomes.
• K3C16 liposomes activated the NLRP3 inflammasome 1.5 fold more than K3C14 liposomes.
• K3C16 liposomes showed significantly lower cytotoxicity in human macrophages than K3C14 liposomes.
• Endo/phagocytosis inhibitors did not block the upregulation of IL-1β release in cells treated with K3C16.
• K3C14 liposomes are endocytosed whereas K3C16 liposomes fuse with the plasma membrane.

## Graphical abstract

**Figure:**
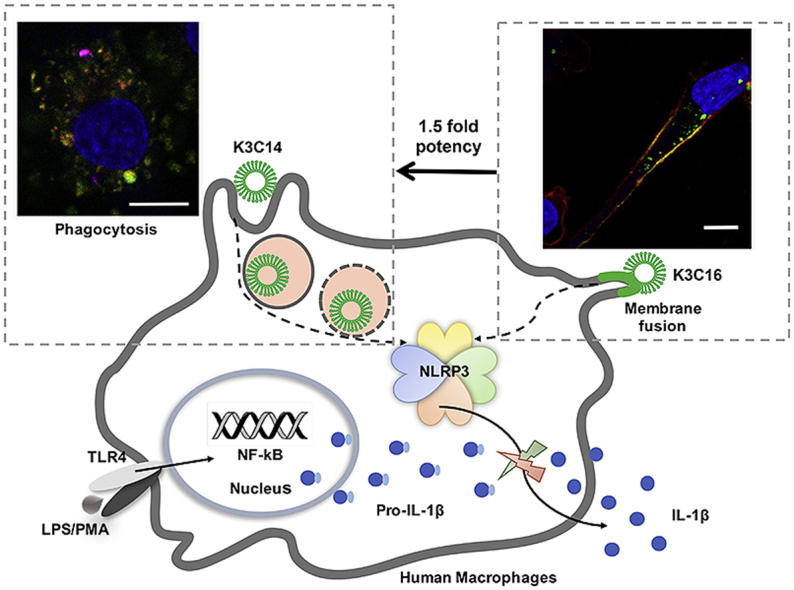

## 1 Introduction

With the growing demand of harnessing human body’s natural defense systems to treat various diseases, nanoparticles such as silica dioxide, titanium dioxide [1] and engineered nanomaterials (ENMs) [2] were investigated in their abilities to activate inflammasomes. The NLRP3 inflammasome, one of the most recognized and characterized multimeric protein complexes, is composed of the NOD-like receptor (NLR) protein NLRP3, the apoptosis-associated speck-like protein containing a CARD (ASC) and pro-caspase-1 [3]. In general, two signals necessitate NLRP3 inflammasome activation; the first one via the toll-like receptors (TLRs) by a wide range of bacterial or viral related molecules induce the production of pro-IL-1β [4]; the second one via various pathways by a broad range of substances such as extracellular ATP via P2X7 receptors [5], the pore forming bacteria toxins nigericin [6], and phagocytic preferential particulate materials such as silica crystals and aluminum salts [7], maturate pro-caspase-1 which cleaves the pro-IL-1β to IL-1β for secretion [8]. Activation of the NLRP3 inflammasomes followed by secretion of IL-1β links the innate immunity to the adaptive immunity via the recruitment of both leukocytes and lymphocytes [9]. Therefore, it is an attractive target for immunotherapy.

Cationic liposomes, a bilayered spherical structure with positively charged outer surface, with high cellular uptake efficiency [10] and consequent endo/lysosomal destabilizing ability [11] have been conventionally employed as non-viral type vectors in the field of gene therapy [12]. To exploit cationic liposomes as immuo-stimulators, Li et al. investigated on a series of lysine type cationic liposomes with alterations on hydrophobic spacer and tail length [13]. The lead assembly, K3C14, bearing lipids with a propyl spacer and ditetradecyl hydrophobic moiety displayed the highest degree of NLRP3 inflammasome activation among the investigated subjects. Previously, lysine type cationic lipids with ditetradecyl or dihexadecyl hydrophobic moiety were reported showing improved transfection efficiency with lower toxicity as lipoplexes [14]. In this study, liposomes of a propyl spacer bearing lysine type lipid with dihexadecyl moietiy, K3C16, were included due to its comparable physical and biochemical traits. It is to our interest to explore its structural effect and mechanism in activating the NLRP3 inflammasomes alongside with K3C14.

Herein, we investigated the cellular uptake mechanism of K3C14 and K3C16, and demonstrated that both liposomes can activate the NLRP3 inflammasomes, however, with K3C16 being 1.5 times more potent than K3C14. Furthermore, we showed that the cellular toxicity of the two liposomes was significantly different. Most importantly, we discovered that in comparison to K3C14 being endocytosed, K3C16 with two hydrocarbon tail length difference entered cells mainly via fusion with the plasma membrane.

## 2 Materials and methods

### 2.1 Materials

The following reagents were purchased: phorbol 12-myristate 13-acetate (PMA) and CP-456773 sodium salt (CRID3) from Sigma-Aldrich (USA); Amicon Ultra 0.5 mL centrifugal filter MWCO 100 kDa, MF-Millipore membrane filters of 0.45 μm and 0.22 μm pore sizes from Millipore Sigma (USA); GM-CSF recombinant human protein, fetal bovine serum (FBS), penicillin-streptomycin, sodium pyruvate (100 mM), GlutaMAX supplement, RPMI 1640 medium, PBS buffer, Pierce LDH cytotoxicity assay kit, cytochalasin D (CytD), Alexa Fluor 488 NHS ester (succinimidyl ester), wheat germ agglutinin Alexa Fluor 555, DRAQ5 fluorescent probe solution (5 mM) and 4% formaldehyde from ThermoFisher Scientific (USA); acridine orange (AO) from Dojindo Laboratories (Kumamoto, Japan); VX765 and lipopolysaccharide (LPS) *Escherichia coli* O111:B4 from InvivoGen (USA); human IL1 beta assay kit from Cisbio Bioassays (France); CD14 microbeads (human) from Miltenyi Biotech (Germany); silica crystals (MIN-U-SIL-15) from US Silica (USA); μ-Slide 8 well ibiTreat from Martinsried (Germany); THP-1 cells from Japanese Collection of Research Bioresource Cell Bank (Japan); CHO-K1 cells and F-12K medium from ATCC (USA).

Human PBMCs were donated from University Hospital Bonn (Germany), and the operation and usage were approved by the local ethics committee in accordance with the Declaration of Helsinki.

### 2.2 Preparation and characterization of liposomes

10 mg of lipids of each type was hydrated in 20 mM HEPES buffer (pH 7.4) at room temperature (r.t.) with vigorous stirring for 4 h followed by extrusion using membrane filters of 0.45 μm and then 0.22 μm pore sizes in an extrusion chamber at 50 °C with the pressure of ∼0.25 pascal (Pa). Size nm in diameter (d.nm) and zeta potential (mV) were measured using Malvern Zetasizer Nano ZS (UK). In short, a 10 μL dispersion of 5 mM liposomes was diluted in 20 mM HEPES buffer to a final volume of 1 mL and was loaded on either a plastic cuvette or a capillary zeta cell for measurements of size and zeta potential, respectively. Three measurements were executed per sample and the mean was reported.

### 2.3 Alexa-488 NHS liposomes conjugation

5% lipid molar concentration of Alexa-488 NHS prepared in DMSO (10 mg/mL) was reacted with primary amines on K3C14 or K3C16 at r.t. in dark for 1.5 h with the highest stirring speed on a stir plate. After completion of the reaction, unreacted Alexa flour was removed using Amicon Ultra-0.5 mL centrifugal filters in a 40-degree fixed angle centrifuge at 14,000×*g* for 5 min. This procedure was repeated twice by filling up the filter with 20 mM HEPES buffer. Then the samples were collected on the filter membrane and the filter was washed several times with a small amount of 20 mM HEPES buffer for the optimal recovery. Lastly, fluorescence intensities of filtrates and samples (dilution factor of 25, 50, 100 and 200) were measured using a fluorescence spectrometer with Gen 5 software in a plate reader. A standard curve of Alexa Fluor 488 ranging from 10 μg/mL to 0.05 μg/mL was used to calculate the reaction rate. The conjugated K3C14_488s and K3C16_488s were characterized with Zetasizer for size, PDI, and zeta potential.

### 2.4 Cell isolation and cell culture

Human PBMCs were isolated by density-gradient centrifugation. Then monocytes were positively selected using CD14 microbeads and subsequently differentiated into macrophages in GM-CSF containing cell culture medium at a density of 2 × 10^6^ cells per mL. Human macrophages were then primed with 1 ng/mL LPS for 2 h prior to stimulation. THP-1 cells were differentiated with 100 nM PMA overnight followed by PBS wash thrice and rested for 1 d. THP-1 cells were primed with 50 ng/mL LPS 2 h before stimulation. Both human macrophages and THP-1 cells were cultured and maintained in RPMI medium supplemented with 10% FBS, 1% sodium pyruvate, 1% L-glutamine and 1% penicillin-streptomycin under standard 37 °C, 5% CO_2_ conditions. CHO-K1 cells were seeded at a cell density of 10^4^/well in 96-well plates and maintained in F-12K medium supplemented with 10% FBS and 1% penicillin-streptomycin under standard 37 °C, 5% CO_2_ conditions.

### 2.5 Immune stimulation

LPS primed human macrophages or THP-1 cells were stimulated with liposome samples (100 μM final concentration used in all *in vitro* assays) over 24 h. 400 μg/mL silica crystals were used as a positive control; LPS primed human macrophages without further stimulation were used as a negative control. For NLRP3/caspase-1 dependency assay, we added NLRP3 inhibitor CP-456773 (5 μM) or caspase-1 inhibitor VX765 (25 μM) 30 min prior to stimulations. For phagocytosis inhibition, 15 μM cytochalasin D was added just after 2 h LPS priming and was kept along with the stimulator. Similarly, an endocytosis inhibitor dynasore (15 μM or 25 μM) was applied for the evaluation. Cells stimulated with liposome samples untreated with cytochalasin D or dynasore were evaluated in parallel. Concentration of IL-1β in supernatants was measured using a HTRF human interleukin beta (IL1β) assay following the manufacturer’s instructions.

### 2.6 Cytotoxicity

Released lactate dehydrogenase (LDH) in culture medium by human macrophages with and without treatment of stimuli after 24 h incubation was measured using the Pierce LDH Cytotoxicity Assay Kit. The assay was carried out according to the manufacturer’s protocol.

### 2.7 Confocal microscopy and fluorescence microscopy

Liposome-cell membrane fusion was observed using a Leica SP2 AOBS confocal laser scanning microscope, 63× water immersion objective. First, 100 μM Alexa-488 labeled liposomes were incubated with LPS primed human macrophages on a μ-Slide 8-well plate (1.5 × 10^5^ cells/well) for 2 h at 37 °C. Then, cells were fixed with 4% formaldehyde for 15 min at 37 °C followed by DPBS wash twice. 5.0 μg/mL Wheat Germ Agglutinin Alexa-555 conjugate was used to stain cell membrane for 10 min at r.t. Lastly, cells were washed twice in DPBS and 2.5 μM DRAQ5 counterstain was added. Liposome-CHO-K1 cell membrane fusion was observed using a Keyence BZ-X series, 20× objective. Simply, 200 μM Alexa-488 labeled liposomes were incubated with 60%–70% confluent CHO-K1 cells on a 96-well glass-bottom plate for 10 min at 37 °C followed by DPBS wash thrice before observation.

### 2.8 Lysosome rupture

PMA differentiated THP-1 cells were treated with 5 μg/mL acridine orange (AO), a lysosomal dye, for 20 min in dark at 37 °C. Subsequently, cells were washed thrice in DPBS and liposome samples prepared in cell culture medium were added and incubated for 20 h. Cells without AO staining and stimulations were served as a positive control (100% lysosomal rupture); cells with AO staining and without stimulations were served as a negative control (0% lysosomal rupture). Prior to flow cytometry, all cell samples were detached from a cell culture plate with 2 mM EDTA, 10–15 min incubation at 37 °C and gentle pipetting. Cell culture medium was replaced with chilled DPBS and all the samples were kept on ice. Flow cytometry and data processing were performed using DB Bioscience FACSAria II cell sorter and DIVA software. R-phycoerythrin (PE) filter channel was selected. Calibration of the flow cytometer was conducted according to the DB manual. Prior to recording the events, the fluorescence intensity was adjusted by changing the strength of voltage; an intensity of about 10^3^-10^5^ was set as the negative control and an intensity of about 10^0^-10^3^ was set as the positive control (P2 gating). Then, about 10,000 events were recorded for all samples. Cells were first gated by SSC-H vs. FSC-H followed by P2 gating (Supplementary Fig. 3). The percent lysosome rupture was calculated and displayed on histogram.

### 2.9 Statistical analysis

Statistical analyses were done using unpaired Ordinary one-way ANOVA multiple comparisons or two-way ANOVA by Prism 7 (Graphpad). P-value style: 0.1234 (not significant), 0.0332 (*), 0.0021 (**), 0.0002 (***), <0.0001 (****).

## 3 Results

### 3.1 Activation of inflammasomes by cationic liposomes in the NLRP3/Caspase-1 dependent fashion

**Fig. 1:**
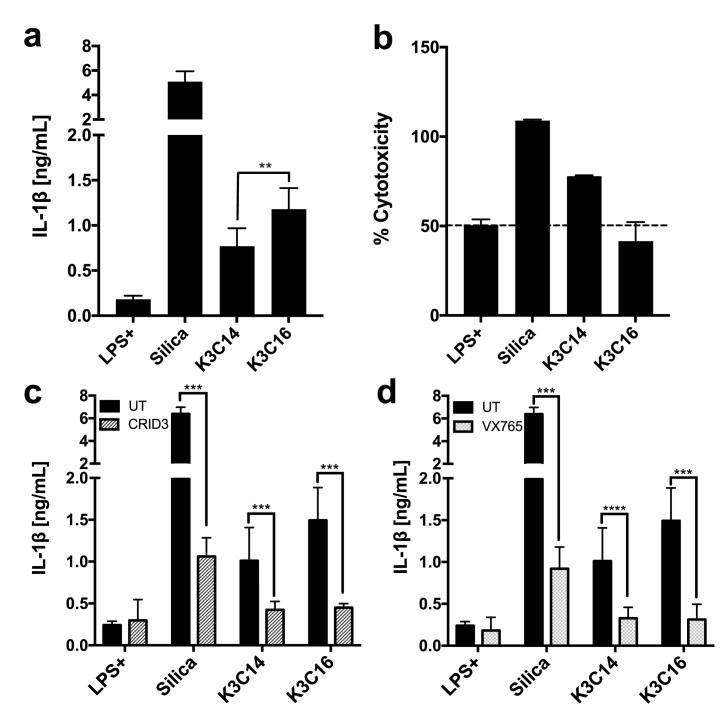
**Activation of inflammasomes by cationic liposomes in the NLRP3/Caspase-1 dependent fashion** (**a**) IL-1β measured from supernatants of LPS primed human macrophages and stimulated by liposomes or silica crystals. (**b**) Toxicity of liposomes or silica crystals to LPS primed human macrophages. Supernatants were collected for LDH cytotoxicity assay. 0% indicates no release of LDH. (**c**) NLRP3 inhibition by treating cells with 5 μM CRID3 (CP- 456773) or (**d**) Caspase-1 inhibition by treating cells with 25 μM VX765 for 30 min at 37 °C, 5% CO_2_ followed by stimuli addition. Cells untreated with stimuli were a negative control. Supernatants were collected after 24 h stimulation for IL-1β and LDH cytotoxicity assays. 100 μM K3C14, K3C16 and 400 μg/mL silica crystals were used in all experiments. Data show mean ± SEM from three donors (**a**) and mean ± SD from one or two donors (**b, c, d**). P-value style: 0.1234 (not significant), 0.0332 (*), 0.0021 (**), 0.0002 (***), <0.0001 (****).

Cationic liposomes of K3C14 and K3C16 were prepared using a simple hydration/sonication method followed by extrusion. The resultant K3C14 and K3C16 liposomes displayed high similarity in particle size, PDI, and zeta potential (Supplementary Table 1). We then evaluated the liposomes’ immunostimulatory strength by checking the secreted IL-1β level in cell supernatants and verified that the NLRP3 inflammasome is the dominant cellular machinery driving the immune response. As shown in Fig. 1a, both liposomes consistently triggered the release of IL-1β with K3C16 exhibiting 1.5-fold higher induction strength than K3C14. Interestingly, K3C16 was barely toxic to cells, which was indicated with a % cytotoxicity below the natural cause of cell death (about 50% cytotoxicity) after 24 h incubation; on the other hand, K3C14 caused nearly 80% cell death (Fig. 1b). With the presence of NLRP3 inhibitor or Caspase-1 inhibitor, CRID3 or VX765, respectively, we demonstrated that both K3C14 and K3C16 triggered IL-1β release was primarily NLPR3 and Caspase-1 dependent (Fig. 1c and d). Correlated scatter plot was showed in Supplementary Fig. 1.

### 3.2 Evaluation on endocytic entry routes

**Fig. 2:**
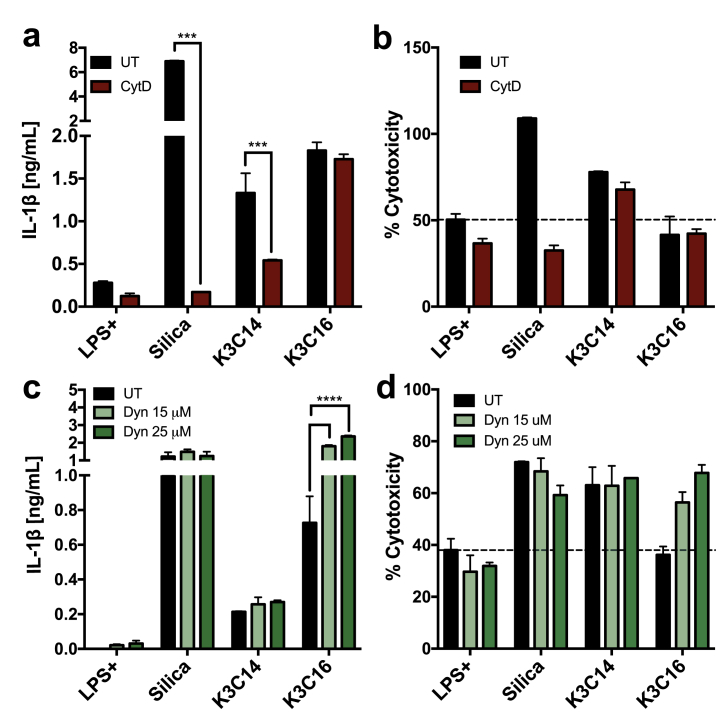
**Evaluation on endocytic entry routes** (**a**) IL-1β measured from supernatants of LPS primed human macrophages, with or without the presence of 15 μM cytochalasin D (CytD), stimulated by liposomes or silica crystals. (**b**) Toxicity of liposomes silica crystals to human macrophages, with or without the presence of 15 μM cytochalasin D. (**c**) IL-1β measured from supernatants of LPS primed human macrophages, with or without the presence of 15 μM or 25 μM dynasore (Dyn), stimulated by liposomes or silica crystals. (**d**) Toxicity of liposomes silica crystals to human macrophages, with or without the presence of 15 μM or 25 μM dynasore. Supernatants were collected after 24 h stimulation for IL-1β and LDH cytotoxicity assay (**a, b, c, d**). 100 μM K3C14, K3C16 and 400 μg/mL silica crystals were used in all experiments. Data show mean ± SD from one or two donors. P-value style: 0.1234 (not significant), 0.0332 (*), 0.0021 (**), 0.0002 (***), <0.0001 (****).

We investigated on the endocytic entry route utilizing two different endocytic inhibitors, cytochalasin D and dynasore. Cytochalasin D is a well-known phagocytosis inhibitor, which acts on blocking the polymerization of the actin filaments. Dynasore, a dynamin inhibitor, inhibits the action of dynamin “nipping” off the endocytic entrapment to form the endocytic vesicle. As shown in Fig. 2a, in the presence of cytochalasin D, release of IL-1β was significantly suppressed in human macrophages stimulated with silica crystals (a positive control) or K3C14. However, cytochalasin D was not effective to block the release of IL-1β from K3C16 stimulated cells. With respect to toxicity (Fig. 2b), silica crystals stimulated cells, normally causing 100% cell death, had shown a complete sabotage of toxicity; K3C14 stimulated cells had shown a similar degree of cytotoxicity with or without the treatment of the phagocytosis inhibitor. In the presence of dynasore (15 μM or 25 μM), IL-1β secretion and cytotoxicity were not affected in silica crystals or K3C14 stimulated human macrophages. Interestingly, an up-regulation of IL-1β secretion was observed in K3C16 stimulated cells (Fig. 2c). In addition, K3C16 stimulated cells in the presence of dynasore displayed an increased cytotoxicity corresponding to the elevated IL-1β secretion (Fig. 2d). Correlated scatter plot was showed in Supplementary Fig. 2.

### 3.3 Lysosome rupture

**Fig. 3:**
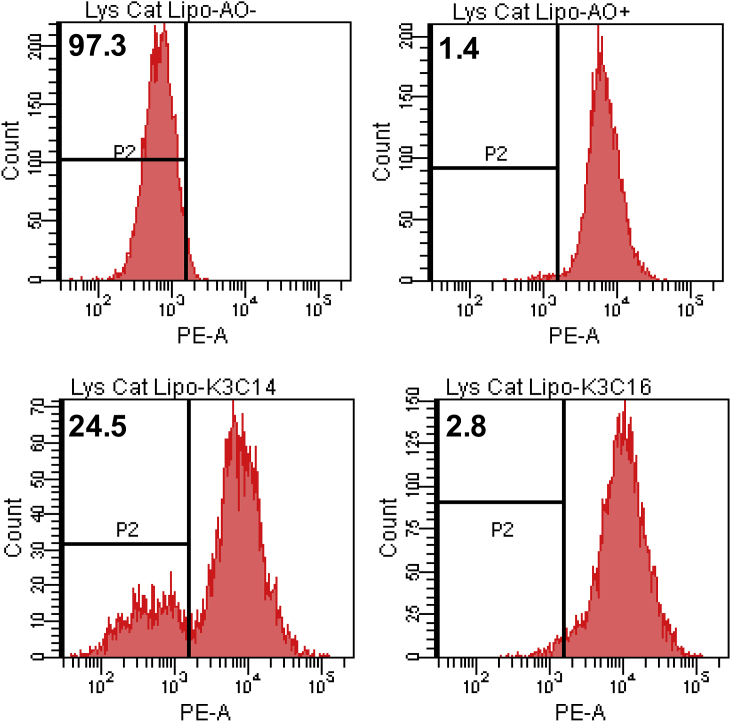
**Lysosome rupture** PMA primed THP-1 cells stained with 5 μg/mL acridine orange (AO) for 20 min were stimulated with 100 μM K3C14 or K3C16 for 24 h. Primed THP-1 cells without AO staining and stimulation were a positive control representing cell with significant lysosome rupture (approximately 10^0^-10^3^). Primed THP-1 cells with AO staining without stimulation were a negative control representing cells without lysosome rupture (approximately 10^3^-10^5^). Representative histograms of P2 gating with PE channel (x-axis) vs Cell Count (y-axis) show the % of cells with significant lysosome rupture. (For gating strategy, see Supplementary Fig. 5).

Upon endocytosis, endo/lysosome compartments entrap cationic particles. As endo/lysosomes further acidify, cationic particles act to rupture the lysosomes and subsequently causing the release of lysosomal enzymes. As a consequence, the release of lysosomal enzymes beckon the assembly of NLRP3 inflammasome [15]. Therefore, we examined the % lysosome rupture caused by K3C14 or K3C16. As shown in Fig. 3, K3C14 stimulated cells had about 24.5% lysosomal rupture, whereas K3C16 stimulated cells barely had lysosome rupture (2.8%).

### 3.4 Observation of membrane fusion

**Fig. 4:**
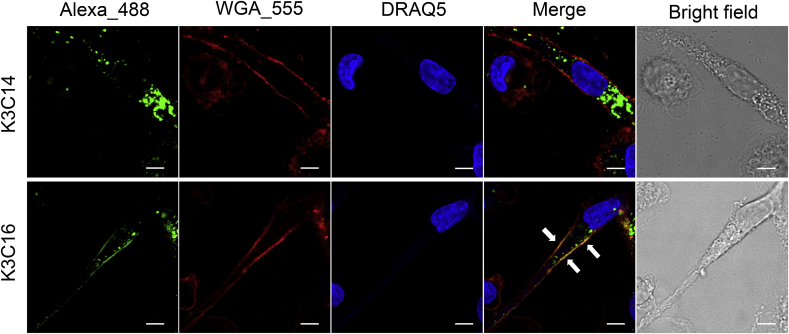
**Observation of membrane fusion** Confocal fluorescence of human macrophages stimulated by 100 μM Alexa 488 conjugated K3C14 or K3C16 for 2 h at 37 °C, 5% CO_2_ and then fixed with 4% formaldehyde solution. Plasma membrane was stained with wheat germ agglutinin Alexa 555 and nuclei were stained with DRAQ5 after fixation. Merged images show the distributions of K3C14_488s (top panel) and K3C16_488s (bottom panel) in the cell. White arrows indicate the colocalization of K3C16 and plasma membrane. Scale bar is 5 μm. Images shown are representative of 2 independent experiments.

Prior to cell imaging experiments, the liposomes of K3C14 or K3C16 was labeled with 5 mol% lipid molar concentration of Alexa 488 (K3C14_488 or K3C16_488, respectively). Comparing with the unlabeled liposomes, K3C14_488 or K3C16_488 showed no significant difference in physical characters (Supplementary Table 1). Microscopic imaging revealed that K3C14_488 liposomes were largely accumulated in the cytoplasm space of the cells (Fig. 4 top panel). Surprisingly, it was observed that K3C16_488 lipids spread along the plasma membrane and overlapped with plasma membrane stain as shown in a merged image (Fig. 4 bottom panel). Additionally, when K3C14_488 liposomes were incubated with LPS primed human macrophages pretreated with lysotracker, colocalization with lysosomes was observed; on the contrary, no lysosome-liposome colocalization was observed in the cells stimulated with K3C16_488 liposomes (Supplementary Fig. 3). To further validate the membrane fusion, K3C14_488 or K3C16_488 liposomes were incubated for 10 min with CHO-K1 cells, a non-phagocytic cell type. It was again shown that K3C14_488 lipids were mainly located in the cytoplasm space, whereas K3C16_488 traced along the plasma membrane of the cells (Supplementary Fig. 4).

## 4 Discussion

In this study, K3C14 and K3C16 cationic liposomes with just two hydrocarbon tail length difference shared high degree of physical similarities in size, PDI and zeta potential. It was demonstrated that both liposomes could trigger IL-1β release primarily through activation of the NLPR3 inflammasomes in LPS primed human macrophages. However, the potency varied between the two; K3C16 had resulted a 1.5-fold higher IL-1β release than K3C14. This finding is consistent with the previous discovery [13] that size, uniformity and surface charge do not have a significant impact on the pharmacological behavior of the lysine type cationic liposomes. Although it was reported by Obata et al. that lysine type cationic liposomes with ditetradecyl or dihexadecyl hydrophobic moiety showed equally lower toxicity in gene therapy studies using cos-7 cells, it was not the case in this study. K3C14 caused a nearly 80% cell death after 24 h incubation, whereas K3C16 caused minimal toxicity to human macrophages. The discrepancy in toxicity suggested that K3C16 is a better candidate than K3C14 as an immunostimulator since high toxicity is most likely to cause adverse effects. More importantly, it implied that K3C14 and K3C16 interacted with cells via different mechanisms.

To confer the cellular uptake mechanism of K3C14 and K3C16 liposomes, endocytosis inhibitors, cytochalasin D and dynasore, were employed. Similar to silica crystals [16], a well-known particulate activates the NLRP3 inflammasome via a phagocytic route, entry of K3C14 liposomes was significantly blocked by a phagocytosis inhibitor, cytochalasin D, but not dynasore. However, toxicity of K3C14 was not reduced, suggesting that K3C14 is intrinsically toxic to cells and/or a minor involvement of other endocytic entry routes. K3C16 liposomes, however, were not taken up by phagocytosis; this was revealed by unsuppressed IL-1β release in the presence of cytochalasin D or dynasore and supported by the fact that no lysosome rupture was detected. Interestingly, an upregulation of IL-1β secretion and an increased cytotoxicity were detected in the cells treated with dyansore and stimulated with K3C16. It was reported by Miyauchi et al. [17] that dynasore inhibited the function of dynamin and surprisingly permitted the mixing of HIV viral lipid to the cell membrane. Hence, the upregulation of IL-1β secretion can be explained by the enhanced membrane fusion event because dynasore not only impairs dynamin-dependent endocytosis, it further impacts the cholesterol recycling to the plasma membrane [18]. A decrease in cholesterol number would increase the fluidity of plasma membrane and therefore would increase the fusion potential of plasma membrane to K3C16 liposomes. As more K3C16 liposomes fused with cells, cytotoxicity increased as a result of robust activation of the NLRP3 inflammasomes.

Lastly, microscopic images clearly shown that K3C14_488 liposomes were located mostly in the cytoplasm space of human macrophages and CHO-K1 cells. It was not surprised to see K3C14 liposomes be taken up by both phagocytes and non-phagocytes. Depending on the cell types, the dominant form of uptake can be different. For instance, it was reported by Satya et al. [19] that caveolae-mediated endocytosis was the cellular uptake mechanism of K3C14 liposome/protein complexes in HeLa cells. K3C16_488 liposomes, to our surprise, overlapped with the cell membranes of human macrophages and same observation was seen using CHO-K1 cells. According to Rejhana et al.’s report [20], there is a trend of increasing fusion potential as the cationic lipid acyl chain gets longer. Because K3C14 and K3C16 share the same lysine head group and as well as nearly identical physical characters, the authors speculate that the disparate biological effects lie in the overall hydrophobicity of the two liposomes. In Sergy et al.’s paper [21], it was shown that a narrow range of hydrophobicity was required for nanoparticles to gain proximity to the cellular membrane and eventually translocate through lipid bilayers. Possibly, hydrophobicity of K3C16 liposomes contributed to achieving a state in which membrane fusion happens when the molecule is about 1 nm close to the cells [22]. Arguably, the gel-to-liquid crystalline phase transition temperature (*T*_*c*_) would be another critical factor determine the cationic liposome fusion potential. As a rule of thumb, membrane rigidity generally increases with elevating the phase transition temperature [23,24]. It was stated in Obata’s report [14] that C16 tail length resulted a higher *T*_*c*_. However, it is important to note that fusogenic potential of C14 and C16 bearing cationic liposomes was not affected when fusing with biomimicking liposomes. Further studies regarding K3C16 membrane fusion mechanism are expected.

Cationic liposomes are widely applied as gene vectors as they can be readily endocytosed by cells. However, current technologies encounter the problem of compromised drug delivery efficiency or high cytotoxicity due to the endocytosis pathway, for example, destabilization of the cargoes by lysosomal enzymes and lysosome dysfunction [11]. Thus, there is a high demand to exploit alternative cell entry route for drugs to reach cytosol. K3C16 liposomes, which fuse with plasma membrane may provide a solution for direct cytosolic drug delivery with low cytotoxicity. Additionally, the NLRP3 inflammsome activation caused by cationic liposomes was previously recognized as a result of lysosome rupture following the endocytosis [13]. In contrast, K3C16 liposomes activated the NLRP3 inflammsomes by fusion with plasma membrane. Although the molecular mechanisms need further investigation, it is promising to apply this type of liposomes as a new type of immunostimulator. Given the likelihood that the NLRP3 inflammsome activation is necessary for conventional adjuvant alum to drive adaptive immune responses [9], K3C16 liposomes are speculated to be good candidates of immune adjuvants with relatively high efficiency in antigen delivery and high potency in immune stimulation. However, these cationic complexes are only proposed for local administration *in vivo*, to restrain the systemic inflammation and prevent opsonization for particle clearance.

In conclusion, we disclosed that K3C14 and K3C16 liposomes sharing the same lysine head group and propyl spacer but alteration on hydrophobic tail length by two hydrocarbons had different immunostimulatory effect (NLPR3 inflammasome activation strength), biocompatibility, and interacted with cells through two distinct routes: K3C14 via endocytosis and K3C16 via membrane fusion.

## Conflicts of interest

E.L. is a co-founder and consultant of IFM Therapeutics. The other authors declare no conflicts of interest associated with manuscript. S.T. is an inventor of JP 5403324 (Waseda U) for the lysine type lipids.
