# Supplementary material for: Membrane fusogenic lysine type lipid assemblies possess enhanced NLRP3 inflammasome activation potency
Source: Biochem Biophys Rep. 2019 Apr 12;18:100623. doi: 10.1016/j.bbrep.2019.100623 (PMC6462779; doi:10.1016/j.bbrep.2019.100623)
Supplement: Multimedia component 2 [file mmc2.docx]

**Supplementary Table 1** Lysine type liposome assemblies, K3C14, K3C16 and Alexa 488 conjugated K3C14, K3C16, were characterized for particle size in diameter (d.nm), polydiversity index (PDI) and zeta potential (mV). Data shown mean ± SEM of 3 independent experiments.

| **Liposome Name** | **Size Distribution (d.nm)** | **Ave. PdI** | **Ave. Zeta Potential (mV)** |
| --- | --- | --- | --- |
| K3C14 | 125±73 | 0.155±0.018 | 44.3±1.84 |
| K3C14_488 | 171±80 | 0.196±0.025 | 43.8±1.18 |
| K3C16 | 117±72 | 0.212±0.006 | 42.0±1.15 |
| K3C16_488 | 122±81 | 0.290±0.019 | 41.2±1.72 |

**Supplementary Figure 1 Activation of inflammasomes by cationic liposomes in the NLRP3/Caspase-1 dependent fashion** (correlated to Figure 1).

**Supplementary Figure 2 Evaluation on endocytic entry routes** (correlated to Figure 2).

**Supplementary Figure 3 Liposome-lysosome colocalization** Confocal fluorescence of human macrophages stimulated by 100 μM Alexa 488 conjugated K3C14 or K3C16 for 2 h at 37˚C, 5% CO_2_ and then fixed with 4% formaldehyde solution. Lysosomes were stained with LysoTracker Red DND-99 for 30 min at 37˚C, 5% CO_2_ prior to stimulation. Nuclei were stained with DRAQ5 after fixation. Merged images show colocalization (orange fluorescence) of K3C14_488 or K3C16_488 (green fluorescence) with stained lysosomes (red fluorescence). Scale bar is 5 µm. Images shown are representative of 2 independent experiments.

**Supplementary Figure 4 Observation of membrane fusion** Live cell imaging, fluorescence (left) merged with bright field (right), of CHO-K1 incubated with 200 μM Alexa 488 conjugated K3C14 or K3C16 for 10 min at 37˚C, 5% CO_2_ followed by DPBS wash thrice. Scale bar is 50 µm. Images shown are representative of 2 independent experiments.

**Supplementary Figure 5 Gating Strategy** First, cell debris were excluded from the population being analyzed (P1 gating). Then, an intensity of about 10^3^-10^5^ was set as a negative control (0% rupture) and an intensity of about 10^0^-10^3^ was set as a positive control (100% rupture; P2 gating). About 10,000 events were recorded for all samples.
